# Near Net Forming Research for Fin-Typed LED Radiator

**DOI:** 10.3390/ma15051736

**Published:** 2022-02-25

**Authors:** Liyong Ni, Shuting He, Yongle Huang, Longjiang Niu, Gaofang He, Wei Peng

**Affiliations:** 1 School of Mechanical and Electrical Engineering, University of Electronic Science and Technology of China Zhongshan Institute, Zhongshan 528400, China; nily@zsc.edu.cn (L.N.); huang874560382@163.com (Y.H.); pengwei@zsc.edu.cn (W.P.); 2 Zhong Shan City Richsound Electronic Industrial Ltd., Zhongshan 528400, China; tinky.he@richsound.com; 3 School of Mechanical Engineering, Shanghai Dianji University, Shanghai 201306, China; 4 Nextorch Industries Co., Ltd., Yangjiang 529931, China; gaofanghe@163.com

**Keywords:** radiator, near net forming, cold forming, cold extrusion

## Abstract

Pure aluminum radiator is the best choice for heat dissipation of various LED products at present. Its forming methods include common extrusion, die casting, forging, etc. Compared with other forming technologies, the LED radiator formed by cold forging has good heat dissipation performance, but there are some disadvantages in the forming process, such as uneven deformation, large material consumption and low die life. The cold forging process of pure aluminum fin-typed LED radiator is analyzed by the finite element method. The calculation results show that equal fillet structure of concave die is improper, leading to serious uneven flow velocity distribution during aluminum forging, inconsistent fin length, and warpage tendency. The gradient fillet structure of concave die is adopted. Numerical simulation and production test show that the gradient fillet structure design can significantly reduce the uneven metal flow. The extruded fins have a uniform length, which is conducive to reducing subsequent machining and production cost.

## 1. Introduction

There are several radiator types, including pure aluminum radiator, aluminum-copper tube radiator, riveted fin-copper tube radiator, phase transformation aluminum radiator. Due to the complex processing technology and difficulties of the latter three methods, they are not of great significance for ordinary products. Pure aluminum radiator is the best choice for heat dissipation of various LED products at present. Its forming methods include extrusion forming, die casting, forging, etc.

Many scholars focused on the production processing and thermal performance of die cast aluminum radiators. Timelli et al. investigated the fatigue reliability of a die cast AlSi11Cu2(Fe) alloy radiator at different pressure amplitudes and thermal exposure [1,2]. Their research object are aluminum alloy heating radiators for domestic use to heat up a room. Lei et al. used investment casting and topology optimization technology to make heat sinks in order to improve the heat transfer effect, but investment casting is unsuitable for the pure aluminum LED radiators because of its low production efficiency [3]. Suresha et al. designed the casted heat sink to eliminate the air entrapping defects by means of FDM [4]. Singh et al. adopted Buckingham’s π approach in the cold chamber die casting process to control the casting defects in the die casting process [5]. The ADC12 material is usually selected for the radiator produced by die casting, but the thermal conductivity is not high at about 96.2 W/(m·K). At the same time, defects such as porosity and shrinkage are easy to appear in the production process, which reduces the heat dissipation performance. Otherwise, the radiator fin produced by this method has a large thickness and heavy product weight [6].

The production method of hot extrusion-assembly radiator is adopted in industry. The fin part and the heat dissipation bottom support plate of the LED radiator are extruded, respectively. Then, two parts are assembled. The radiator manufactured by this method has large contact thermal resistance. The hot extrusion method could be a good choice for products of constant cross-section, such as CPU heat sinks, thin-walled aluminum alloy profile, and multi-port tubes [7,8,9].

The cold extruded radiator is manufactured by plastic deformation of a solid aluminum block in the die under high pressure at room temperature. The radiator manufactured by this technology reduces the contact thermal resistance, and the thermal coefficient of the radiator is 226 W/(m·K), higher than that of the radiator produced by die casting [10].

**Figure 1:**
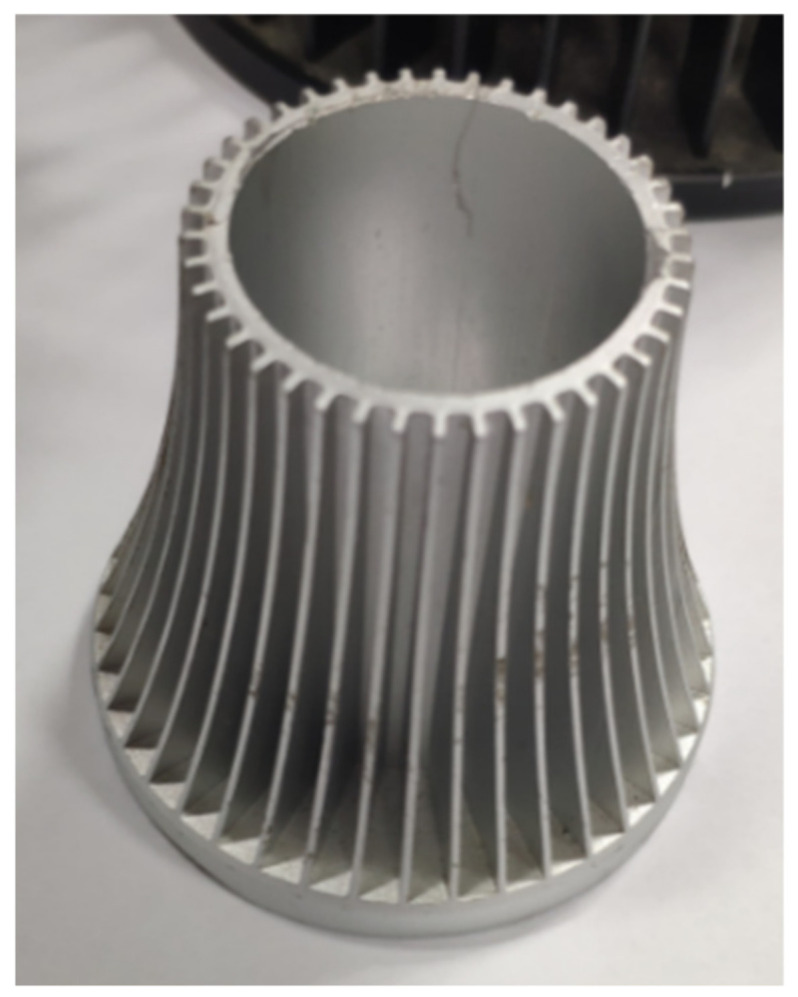
Cold extruded radiator.

The fin thickness of die casting aluminum radiator is generally thick, while the fin thickness of cold forging radiator can be very thin, whose structure is beneficial to increase the number of fins and the heat dissipation area. Figure 1 shows an example of a cold extruded radiator. The radiator has even fin height and thin thickness.

**Figure 2:**
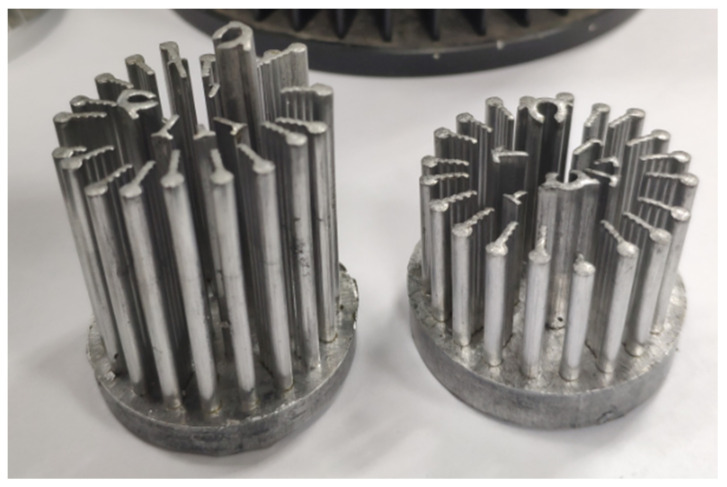
Poor cold forgings of LED radiator.

Some enterprises have adopted the cold forging technology in actual production, but there are still some problems such as uneven cold forging, large material loss, and low die life. Figure 2 shows poor cold forgings of LED radiators. The radiators have uneven fin heights. Successive machining is needed to obtain even height. Near net forming technology is necessary to obtain accurate geometry as far as possible, reducing material wasting and machining cost.

Many scholars have done a lot of research work on the plastic forming process of LED radiator, including cold heading, cold forging, cold extrusion, etc., but their studies focus mainly on hot extruded aluminum profile. However, there are few reports on the cold forging process of a radiator.

Xuemei et al. discussed the structure and key manufacturing technology of the extrusion die for sunflower aluminum alloy radiator [11]. Rurong et al. introduced the extrusion die structure of a plug-in high-density tooth aluminum radiator [12]. Weiping et al. introduced the characteristics, classification, and material selection of aluminum radiators [13]. Hongbo et al. used HyperXtrude to conduct numerical simulation and die structure optimization for extrusion of high-density tooth radiator profile [14]. Biao et al. carried out a numerical simulation on the extrusion of the aluminum profile of radiator [15]. Hwang and Shen carried out finite element analysis on the hot extrusion process of CPU aluminum profile radiator and optimized the structural parameters of the extrusion die [16]. However, what they introduce is the hot extrusion aluminum profile technology.

Hongliang et al. optimized the cold forging process parameters of a cylindrical LED radiator by using FORGE-3D software [17]. With the help of DEFORM-3D finite element software, Jianguang et al. simulated the extrusion process of a pin-rib radiator, and analyzed the effects of friction coefficient, die fillet radius, and punch size [18]. However, compared with a fin radiator, the materials consumption of a cylindrical radiator is relatively large, and the heat dissipation area is relatively small.

Compared with cylindrical radiators, fin-typed radiators have a larger specific surface area and higher heat dissipation effect under the condition of the same mass of raw materials. However, the thinner the fin thickness is, the more difficult it is to deform. In practice, there is insufficient filling or a too large difference in fin heights, resulting in increased scrap rate or too large cutting allowance. In order to overcome the above problems, this paper takes Al 1070 fin-typed LED radiator as an example and uses rigid plastic FEM to predict metal flow and forming quality and experimental verification so as to provide the basis for the development of the said cold forgings.

## 2. Research Methodology

With regard to the uneven length of fins, the cold forging die structure of pure aluminum fin-typed LED radiators was modified. FEM method based on rigid-plastic theory was adopted to predict the material flow. Pilot production was conducted to verify the simulation results.

**Figure 3:**
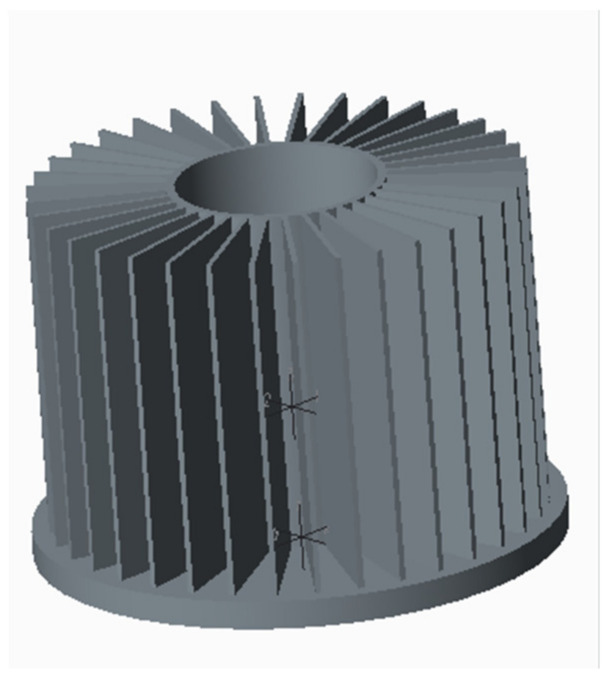
Integral fin-typed radiator.

The reference part is shown in Figure 3. The material of the studied radiator is AL 1070. The diameter is 92 mm, the height of the base is 5 mm, and the height of the hollow column in the center is 60 mm. The fin width is 24 mm, the fin thickness is 1 mm, the fin number is 36, the product weight is 271 g, and the heat dissipation area is 1241 cm^2^.

During the actual production process, the diameter of the billet must be slightly smaller than the diameter of the die for the positioning convenience. The billet diameter is chosen as 91 mm. The billet height is 19.68 mm, according to volume invariance principle.

**Figure 4:**
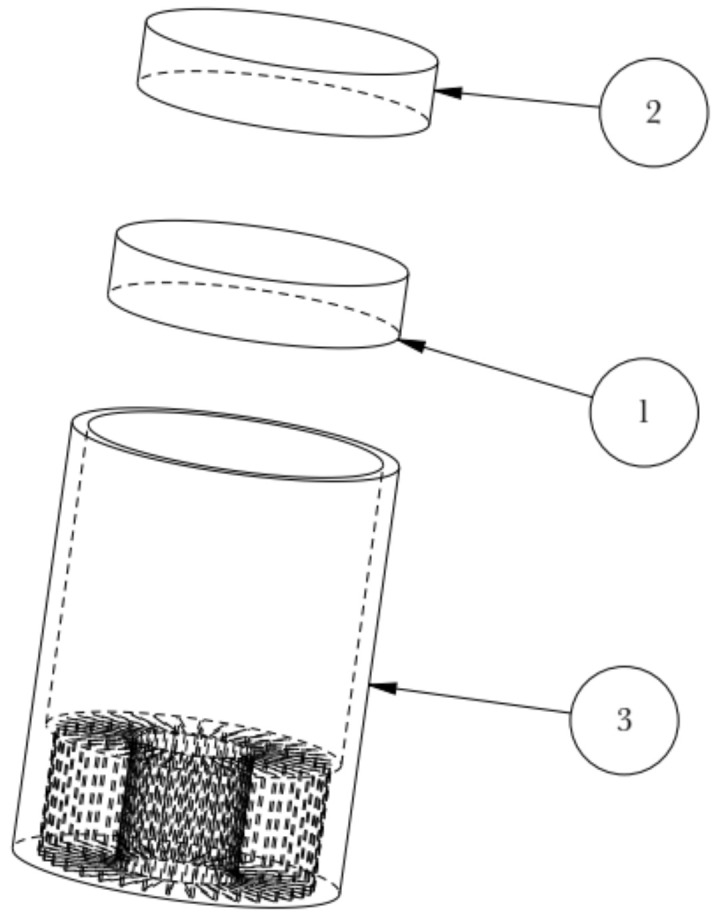
Schematic diagram of relative position relationship of die working parts. 1. Billet; 2. Punch; 3. Concave die.

**Figure 5:**
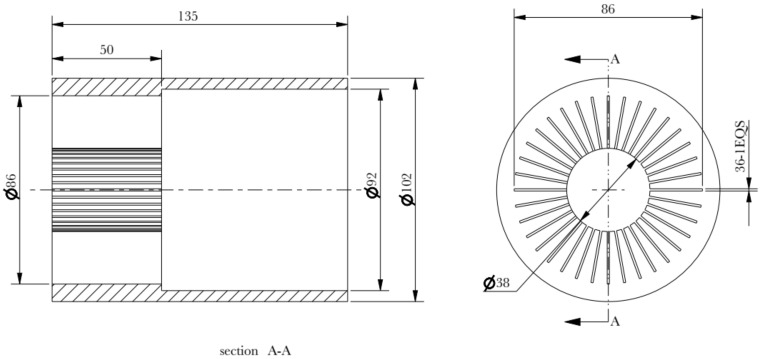
Geometric model of the concave die.

The explosion schematic diagram of the assembled die is shown in Figure 4. The geometric model of the concave die is shown in Figure 5. The wall thickness of the concave die is 5 mm, and the inner diameter is 92 mm. The fin thickness of the concave die is 1 mm. The concave die depth is 50 mm.

The closer the diameter of the punch is to the diameter of the die, the less flash will occur in the subsequent cold forging process. Therefore, the set diameter of the punch is 91.5 mm.

In the simulation of the cold forging process, the rigid plastic finite element method is adopted. The elastic deformation of the billet is ignored. The billet is set as a rigid plastic body. The punch and concave die are set as rigid bodies. The concave die is fixed. The extrusion speed of punch is 10 mm/s. The friction coefficient between die and billet is 0.12. The temperature of billet and die is 293 K. The free meshing method is adopted. The original number of elements is 80,000, and the step size is set to 0.0743 mm.

The basic equations to be satisfied during deformation are as follows:

(1) Equilibrium differential equation:
  (1) $$\sigma_{ij,j}=0$$
(2) Constitutive equation (stress–strain rate relationship):
  (2) $${\sigma^{\prime }}_{ij}=\frac{2\bar{\sigma }}{3\dot{\bar{\varepsilon }}}{\dot{\varepsilon }}_{ij}$$
  where $\dot{\bar{\varepsilon }}$ is equivalent strain rate, $\dot{\bar{\varepsilon }}=\sqrt{\frac{2}{3}{\dot{\varepsilon }}_{ij}{\dot{\varepsilon }}_{ij}}$ ; $\bar{\sigma }$ is equivalent stress, $\bar{\sigma }=\sqrt{\frac{3}{2}{\sigma^{\prime }}_{ij}{\sigma^{\prime }}_{ij}}$.
(3) Geometric equation (Strain rate–velocity relationship):
  (3) $${\dot{\varepsilon }}_{ij}=\frac{1}{2}\left(u_{i,j}+u_{j,i}\right)$$
(4) Volume invariant condition:
  (4) $${\dot{\varepsilon }}_{v}={\dot{\varepsilon }}_{ij}\delta_{ij}={\dot{\varepsilon }}_{ii}={\dot{\varepsilon }}_{11}+{\dot{\varepsilon }}_{22}+{\dot{\varepsilon }}_{33}=0$$
(5) Boundary condition:
  Mechanical boundary conditions on forced surface $S_{F}$
  (5) $$\sigma_{ij}n_{j}=F_{i}$$
  Velocity boundary condition on velocity surface $S_{u}$
  (6) $$u_{i}={\bar{u}}_{i}$$
  The material in the deformation zone must meet the yield conditions
  (7) $$f=\sqrt{{J^{\prime }}_{2}}-K=0$$
  where ${J^{\prime }}_{2}$ is second invariant of stress deviator tensor and K is shear yield limit.

Equations (1) and (2) are the basic equation for the finite-element formulation. The iteration methods adopted for solving the nonlinear equations are Newton–Raphson and the direct iteration methods. The direct iteration method is used to generate a good initial guess for the Newton–Raphson method, whereas the Newton–Raphson method is used for speedy final convergence.

## 3. Results and Optimization

### 3.1. Material Flow

**Figure 6:**
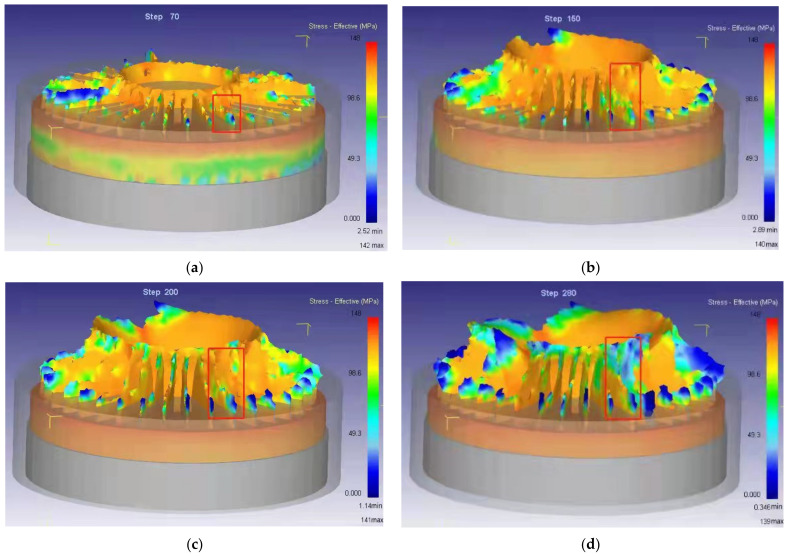
Stress distribution and flow of fins during the forming process. (**a**) Step = 70; (**b**) Step = 150; (**c**) Step = 200; (**d**) Step = 280.

Figure 6 shows the stress state of fins during the forming process. It can be clearly seen that the stress changes dramatically during the contact between the fin and the concave die, and when the step = 280, the stress distribution is uneven, the forming is not full, and the fin length is inconsistent. The billet geometry is far from that in Figure 3. It means that the conventional die structure cannot realize the net forming.

### 3.2. Strutre Modification of Concave Die

**Figure 7:**
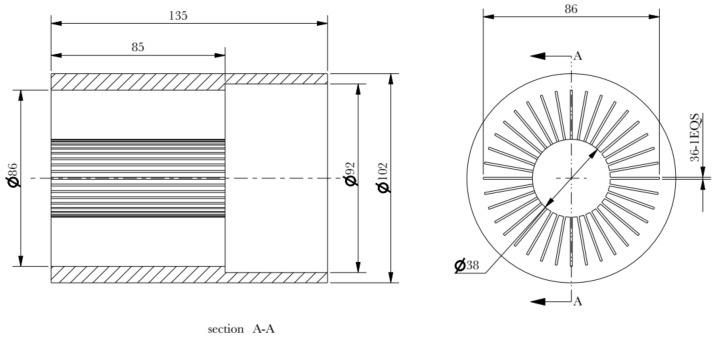
Concave die of modified through-hole depth.

In order to reduce the bending of the fin column, the through-hole depth of the die is modified longer than the longest length of the required fin. Since the fin height is 60 mm, the through-hole depth is modified to 85 mm, as shown in Figure 7.

**Figure 8:**
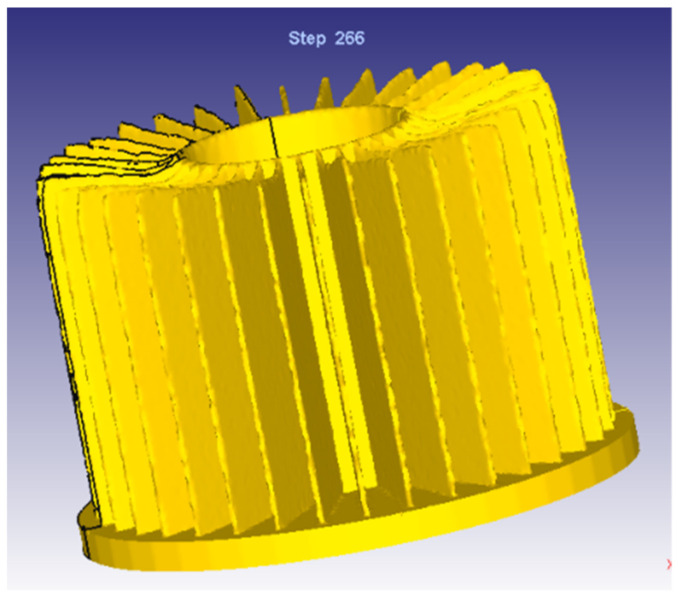
Radiator after firstly-modified die structure.

Adopting the same process parameters and modifying the through-hole depth of the die, the radiator, as shown in Figure 8, is obtained. As can be seen from Figure 8, the whole geometry was improved very much and near to that in Figure 3. It means that the modification of the through-hole depth of concave die is effective. However, the formed end face is not flat enough, so the die design needs to be improved again.

During the extrusion process, the velocity in the middle part of the billet is higher than that in the edge part. This will result in different fin heights. In order to correct this flow velocity difference, the concave die is slightly gradually rounded.

**Figure 9:**
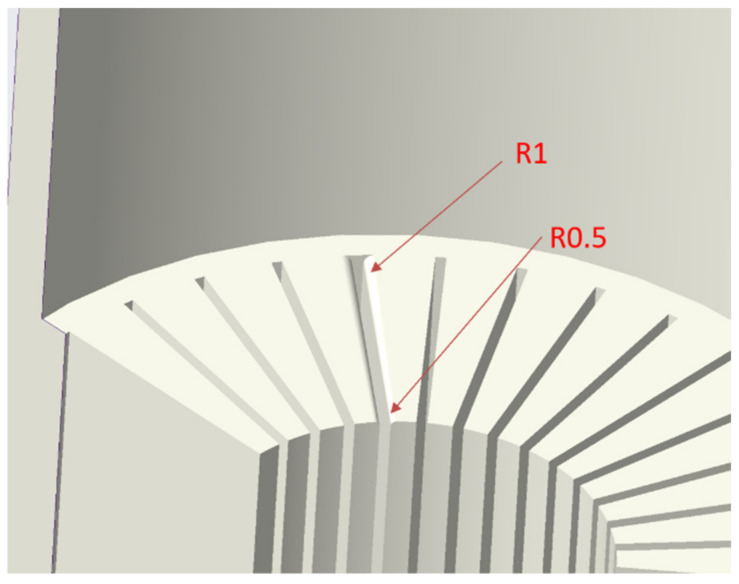
Fillet gradient design of concave die.

Kwan et al. investigated the influence of die fillet design on extruded fin lengths of straight-fin heat sink extrusion [19]. With reference to their results, the fillet of the concave die could be redesigned. The fillet of the concave die reduces the contact area between the die and the billet and changes the flow rate of the billet in the extrusion process. The fillet at the outer circumference of the concave die is larger than that of the center of the die. As shown in Figure 9, the fillet of the concave die is set as 0.5 mm at the center and 1 mm at the outer circumference.

### 3.3. Simulation and Pilot Production after Modification of Mold Structure

**Figure 10:**
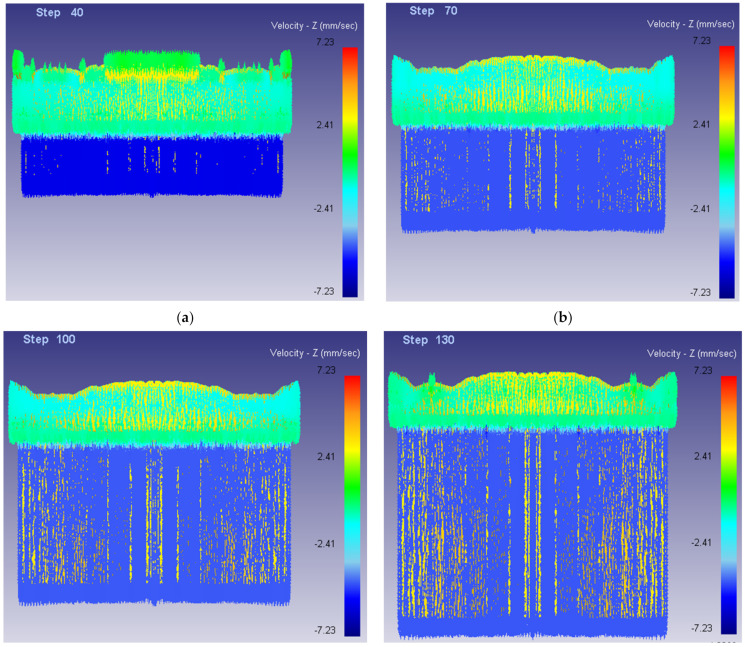
Flow velocity diagram of billet along Z direction (Front view). (**a**) Step = 40; (**b**) Step = 70; (**c**) Step = 100; (**d**) Step = 130.

Figure 10 shows the flow velocity distribution along the Z direction of the billet of the second improved scheme. As shown in Figure 10, the flow velocity at the center is basically the same as that at the periphery of the die, and there is no flow anomaly in the flow process.

**Figure 11:**
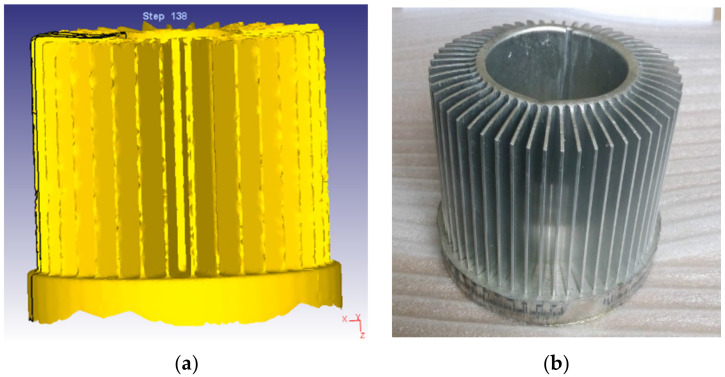
Comparison of simulation and pilot production. (**a**) Predicted geometry based on FEM; (**b**) Pilot production.

According to the optimized structure, the mold is processed, and the billet with a smooth and clean surface is selected during actual processing. The billet is dry lubricated before extrusion, the slider speed is set to 10 mm/s, the die surface is cleaned and put in the billet, and lubricating oil is applied on the surface. Figure 11a shows the radiator after secondly modified die structure based on FEM. Figure 11b shows the pilot production according to the modified die structure. It can be seen from Figure 11 that the radiator is well formed and achieves a flat end face. It proves that the gradual fillet plays a good role in controlling the flow velocity of the material.

Table 1 gives the results of conventional die design and optimal structures. It can be seen from Table 1 that conventional die design leads to warpage tendency and large maximum difference of fins lengths. Numerical simulation and pilot production for optimal structures show that there is no warpage and less difference of fin lengths. Their agreement shows that the gradient die fillet design can improve the forming quality.

## 4. Conclusions and Discussion

There is large heat resistance in the radiators produced by the extrusion–assembly method, reducing their heat dissipation efficiency. In addition, there are tiny porosities in the radiators produced by the die casting method, reducing their heat dissipation efficiency, too. On the contrary, there is no large contact heat resistance or tiny porosities in the products by cold extruding. Compared with the parts made by other methods, the cold extruded radiators have dense structures. The thermal conductivity of the radiator formed by Al 1070 cold forging is 226 W/(m·K), higher than that of the radiators produced by die casting.

Taking fin-type Al 1070 radiator as an example, the extrusion process of radiators is analyzed. The traditional die structure adopts an equal fillet design. FEM software was adopted to analyze the metal flow and the resulting deformation geometry for the said structure, based on the rigid plastic theory. The numerical simulation calculation results show that the said design is improper, leading to serious uneven flow velocity distribution of aluminum in the extrusion die, inconsistent fin lengths and prone to warpage deformation. Some defects, such as inconsistent fin length, could be eliminated by machining, but there will be large material costs. Warped products have to be wasted.

In order to reduce the flow velocity difference of materials during extruding, a gradient fillet design was adopted. Considering that the metal flow velocity near the axis is larger than that at the periphery, the fillet radius near the axis is slightly smaller, increasing the flow resistance. The numerical simulation was conducted for the new design under the same forming parameters. The simulation results show that near equal fin length can be obtained, and the filling quality is satisfying.

Pilot production was conducted according to the new design. Simulation and experimental results can agree with each other, showing that the gradient die fillet design can avoid the significant uneven metal flow rate in the die and improve the forming quality.

## Figures and Tables

**Table 1:** Results of conventional die design and optimal structures.

	Conventional Die Design	FEM-Modifying the Through-Hole Depth	FEM-Modifying the Through-Hole Depth and Gradient Fillet	Pilot Production
Warpage	YES	NO	NO	NO
Maximum difference of fin lengths(mm)	>6	0.8	0.2	0.5

## Data Availability

Not applicable.
